# Gleanings

**Published:** 1897-06

**Authors:** 


### GLEANINGS.

Electric-tape is highly recommended as an excellent material for securing in place the bandages on the wounded extremities of dogs. It can be obtained at most electrical-supplies stores.

Dr. R. W. Finlay, of New York, in answer to a correspondent, says osteoporosis is a non-inflammatory disease, more prevalent among males than females, rarely seen after five years of age, and is not contagious.

The average quantity of water consumed daily by six cows varied from 70 to 140 pounds each. These were stabled cows, and for every pound of milk produced, four pounds of water were consumed. In this experiment the cows which gave the richest milk consumed the largest quantities of water.

The *Veterinary Journal* recommends as a good disinfectant one half-ounce of corrosive sublimate and one fluidounce of hydrochloric acid in three gallons of water. It is suggested as a precautionary measure, to avoid its being mistaken and used internally, to add five grains of commercial writing blue or ordinary violet writing-ink, to give it a conspicuously distinguishing character; it should be applied with a whitewash-brush on flat or smooth surfaces, and with a syringe in hidden parts. After remaining over night on stalls, feed-racks, or boxes, these should be washed off with warm water to remove the mercury.

The wireworm (*Strongylus contortus*) is causing losses among the sheep in Australia.

The arseniate of iron and strychnine, in combination with iodide of potassium, has recently been brought forth as efficient treatment for horses whose respiratory organs have become the seat of such changes as to produce roaring, whistling, and emphysema.

